# Altered Patterns of Functional Connectivity and Causal Connectivity in Salience Subnetwork of Subjective Cognitive Decline and Amnestic Mild Cognitive Impairment

**DOI:** 10.3389/fnins.2020.00288

**Published:** 2020-04-21

**Authors:** Chunting Cai, Chenxi Huang, Chenhui Yang, Haijie Lu, Xin Hong, Fujia Ren, Dan Hong, Eyk Ng

**Affiliations:** ^1^School of Informatics, Xiamen University, Xiamen, China; ^2^Department of Radiation Oncology, Zhongshan Hospital of Xiamen University, Xiamen, China; ^3^College of Computer Science and Technology, Huaqiao University, Xiamen, China; ^4^School of Mechanical and Aerospace Engineering, Nanyang Technological University, Singapore, Singapore

**Keywords:** subjective cognitive decline, amnestic mild cognitive impairment, salience network, functional connectivity, causal connectivity

## Abstract

The subjective cognitive decline (SCD) may last for decades prior to the onset of dementia and has been proposed as a risk population for development to amnestic mild cognitive impairment (aMCI) and Alzheimer disease (AD). Disruptions of functional connectivity and causal connectivity (CC) in the salience network (SN) are generally perceived as prominent hallmarks of the preclinical AD. Nevertheless, the alterations in anterior SN (aSN), and posterior SN (pSN) remain unclear. Here, we hypothesized that both the functional connectivity (FC) and CC of the SN subnetworks, comprising aSN and pSN, were distinct disruptive in the SCD and aMCI. We utilized resting-state functional magnetic resonance imaging to investigate the altered FC and CC of the SN subnetworks in 28 healthy controls, 23 SCD subjects, and 29 aMCI subjects. In terms of altered patterns of FC in SN subnetworks, aSN connected to the whole brain was significantly increased in the left orbital superior frontal gyrus, left insula lobule, right caudate lobule, and left rolandic operculum gyrus (ROG), whereas decreased FC was found in the left cerebellum superior lobule and left middle temporal gyrus when compared with the HC group. Notably, no prominent statistical differences were obtained in pSN. For altered patterns of CC in SN subnetworks, compared to the HC group, the aberrant connections in aMCI group were separately involved in the right cerebellum inferior lobule (CIL), right supplementary motor area (SMA), and left ROG, whereas the SCD group exhibited more regions of aberrant connection, comprising the right superior parietal lobule, right CIL, left inferior parietal lobule, left post-central gyrus (PG), and right angular gyrus. Especially, SCD group showed increased CC in the right CIL and left PG, whereas the aMCI group showed decreased CC in the left pre-cuneus, corpus callosum, and right SMA when compared to the SCD group. Collectively, our results suggest that analyzing the altered FC and CC observed in SN subnetworks, served as impressible neuroimaging biomarkers, may supply novel insights for designing preclinical interventions in the preclinical stages of AD.

## Introduction

Alzheimer disease (AD) is a chronic neurodegenerative disorder presented in elderly individuals with conspicuous decline in cognitive deterioration and lapse of memory (Huang et al., 2019; Wessels et al., 2019; Zhang et al., 2019). As one of the phases between normal aging and dementia, amnestic mild cognitive impairment (aMCI) subjects have a 10–15% possibility of developing into AD per year (Petersen et al., 2002; Yang et al., 2017). Subjective cognitive decline (SCD) is the stage referring to the elderly subjects that can last for decades earlier than the onset of dementia when persons subjectively complain of memory impairment without corresponding objective clinical manifestations, while person’s scores are in the normal scope through standardized neuropsychological tests (Huang et al., 2018a; Funaki et al., 2019; Yu et al., 2019). Furthermore, converging evidence suggests that SCD poses risk for developing into MCI and AD, although it may likewise be of early preclinical stages of other neurodegeneration diseases (Berger-Sieczkowski et al., 2019; Caillaud et al., 2019). Thus, it stands to reason that SCD can be utilized in conjunction with aMCI to explore the mechanism of the early phases of AD and to detect it timely.

Numerous authors have applied resting-state functional magnetic resonance imaging (rs-fMRI) as one of the principal means to clarify the cognitive mechanism of AD (Yang et al., 2017; Marchitelli et al., 2018; Passamonti et al., 2019). Besides, the brain network researches relying on rs-fMRI serve the purpose of revealing the mechanism of neural activity in the brain, which have also important application value and significance in exploring the pathogenesis of AD (Donofry et al., 2019; Lee J. et al., 2019). Among them, plentiful works have been examined by scholars involving in the relationships between salience network (SN) and other networks in the brain (Fredericks et al., 2019; Lee S. E. et al., 2019). More specifically, the SN, which is typically involved in detecting stimulus salience, is a large-scale brain network within the human brain (Cai et al., 2019). Anatomically, it can be spilt into anterior SN (aSN) and posterior SN (pSN) and is primarily anchored in frontoinsular cortices and dorsal anterior cingulate cortex (dACC) (Menon and Uddin, 2010). Recent large-scale works of literature point at the altered patterns of FC and causal connectivity (CC) between SN and other networks. A published study has confirmed that individuals with AD exhibit decreased FC within and between the default mode networks (DMNs) and SN in comparison with healthy controls (HCs) (Liu et al., 2019). Additionally, patients with MCI showed increased FC in the right insula lobule (IL) and claustrum within the SN when compared to the HC group. Similarly, for the aMCI subjects, the FC of the SN-centered model [includes SN, DMN, executive control network (ECN)] is impaired compared to the patients with AD, and these alterations in SN-centered model may result in a decline in cognitive disorder (Aguirre et al., 2019). The basic idea of Granger causality analysis (GCA) is based on multiple linear regression to explore whether there is a causal relationship between the two time series; it helps to accurately forecast the current value of another series and is widely utilized to brain science research field (McBride et al., 2015; Xue J. et al., 2019). A recent study using GCA to analyze CC patterns of aMCI has revealed that CC alterations observed in the SN, ECN, and DMN networks may be regarded as impressible neuroimaging biomarkers for the preclinical intervention and detection of aMCI (Zhang et al., 2019). Former investigation has also revealed that pathological alterations existed in the CC of dACC within SN of AD (Petersen et al., 1985). So far, the majority of SN studies have almost focused on the SN network or between SN and other networks, yet very few researches have previously examined whether CC and FC based on SN subnetworks can be used as neuroimaging biomarkers for identifying aMCI and SCD and to explore how the altered regions of FC and CC relate to cognitive function.

Herein, the objective of our work is to analyze the disruptions observed in FC and CC of SN subnetworks for SCD and aMCI. We hypothesized that there be distinct alterations of the FC and CC in SN subnetworks, and they might be regarded as sensitive neuroimaging markers.

## Materials and Methods

### Participant

Data recruited in this article were acquired from the second phase of the Alzheimer’s Disease Neuroimaging Initiative (ADNI-2) database^1^. ADNI-2’s primary goal is to focus on finding biomarkers of cognitive impairment and measures of outcome. ADNI-2 was announced and implemented in 2011 and began with a $67 million foundation. Furthermore, it lasted about 5 years. To investigate the gap between the HC and MCI, patients with SCD were included in ADNI-2 for the first time; detailed descriptions of ADNI-2 can be found in www.adni-info.org. Subjects with HC (*n* = 28), SCD (*n* = 23), and aMCI (*n* = 29) were recruited in the present work. Additionally, five individuals were excluded because of excessive head motion (we controlled cumulative translation or rotation > 1.5 mm or 1.5°, *n* = 3) and quality control in normalization (*n* = 2). Ultimately, a total of 75 subjects were recruited, comprising 27 HC, 20 SCD, and 28 aMCI subjects.

### MRI Data Acquisition

All participants recruited in our work underwent rs-fMRI of 3.0-T Philips Medical Systems (Amsterdam, Netherlands) scanner. The echo-planar imaging sequence contained 140 volumes, and the subjects were separately required to lay subjects on their back, with eyes closed, avoid mentally active brain, and maintain head position during data acquisition. The specific parameters of the scan were as follows: each subject contains 140 time points, flip angle (FA) = 80°, matrix = 64 × 64 × 48, voxel size = 3.31 × 3.31 × 3.31 mm^3^, repetition time (TR) = 3,000 ms, echo time (TE) = 30 ms, slice thickness = 3.3 mm. T1-weighted image volumes were obtained by using magnetization-prepared rapid gradient-echo sequence (Chen et al., 2016), and the parameters were as follows: matrix = 256 × 256 × 170, slice thickness = 1.2 mm, acquisition plane = sagittal, TE = 3.16 ms, TR = 6.81 ms, voxel size = 1 × 1 × 1.2 mm^3^, FA = 9°. All the data involved in this article are universally available to the scientific community.

### Data Pre-processing

For rs-fMRI data, Resting-State fMRI Data Analysis Toolkit plus (RESTplus)^2^ was applied for data pre-processing, which is based on MATLAB2012a^3^ and Statistical Parametric Mapping (SPM12)^4^. Pre-processing for rs-fMRI data involved the following steps: the first five volumes in 140 volumes for each subject were removed for possible instability of rs-fMRI signal, and the remaining 135 points in time were corrected for controlling time differences between slices and head motion effects of volumes. Cumulative translation of more than 1.5 mm or angular motion of more than 1.5° was excluded. Next, normalization was adopted to register the original space to the Montreal Neurological Institute (MNI) space by T1 images to mitigate the differences in brain structure between different individuals. Then, the normalized brain volumes were smoothed using Gaussian kernel of 6 × 6 × 6 full width at half maximum in order to reduce individual variations. Following this, nuisance variables, such as six head motion parameters, global mean signal, white matter signal, and cerebrospinal fluid signal, were severally removed to reduce the effect on the dependent variable (Fox et al., 2009; Huang et al., 2018b). Finally, to control noise interferences such as heartbeat and breathing, the subjects’ brains generated in the previous step were filtered at 0.01–0.08 Hz.

### Statistical Analysis

The distinctions between the HC, SCD, and aMCI groups of demographic and neurocognitive data were estimated by employing analysis of variance (ANOVA) and the *χ*^2^-test within the Statistical Package for the Social Sciences (SPSS) software version 22.0 (IBM, Armonk, NY, United States), and then *p* < 0.05 was set to indicate significant difference in our work.

Comparison and analysis for differences between HC, SCD, and aMCI groups, one-way ANOVA, implemented in the Data Processing and Analysis for Brain Imaging (DPABI)^5^ software, was performed through voxel-by-voxel way within the brain mask after regression of age and gender covariates. As suggested in former research, the false-positive rate can be effectively controlled for multiple comparisons using the non-parametric permutation test at the cluster level (Winkler et al., 2016). Here we adopt 1,000 permutation times, and a cluster size > 30 voxels (810 mm^3^) was set as the significant cluster. Besides, the significance level was set at 0.05 in the permutation test process. The two-sample *t*-test was employed to calculate differences between two groups within the mask generated by ANOVA. Previous study has identified that the non-parametric permutation test with Threshold-Free Cluster Enhancement (TFCE) can strike a good and strict balance between family-wise error rate and reliability (Chen et al., 2018). Consequently, permutation tests with TFCE, implemented in PLAM within DPABI, were utilized to perform multiple comparisons in this work, and then a cluster size > 10 voxels (270 mm^3^) was adopted as the significant cluster, and the significance level was set at 0.05 (Xue C. et al., 2019).

### Independent Component Analysis

Independent component analysis (ICA) is a data-driven and robust analysis technique for separating statistically independent signal sources, which is desirable in exploring neuroimaging data (Beckmann, 2012). Based on former researches, we aimed to use GIFT toolbox (v4.0b)^6^ and the infomax algorithm to obtain SN subnetwork components of all subjects (Duc et al., 2019; Liu et al., 2019). To obtain more accurate aSN and pSN components, we first split the data into 20, 25, 30, 35, 40, 45, and 50 components. It is noted that the aSN and pSN templates were acquired by pre-decessors’ research (Shirer et al., 2012). A previous study has shown that the component with the highest spatial correlation value is most similar to the template (Cai et al., 2017), and then mean spatial maps of each component were severally utilized to run spatial correlations with SN subnetworks templates (Aguirre et al., 2019). Furthermore, a former study has reported that visual recognition of components through observation and comparison of three researchers was the same as or better than machine recognition approaches (Cherubini et al., 2009). Taken together, we obtained 40 independent components through the collaboration of three researchers and ICA for subsequent analysis, and the components most corresponding to aSN and pSN were 34 and 14, respectively. Since the intensity values in the ICA spatial map have been converted to *z*-values, we directly performed one-sample *t*-test (*p* = 0.05, TFCE-FWE corrected, cluster size > 10 voxels) of all subjects to ascertain the aSN and pSN components, respectively.

### Functional Connectivity Analysis

Two types of masks, comprising aSN and pSN masks, were obtained according to the ICA analysis. Then, mask-based FC analysis was carried out to examine the alteration patterns between SN subnetworks and the whole brain. Following this, Fisher *r*-to-*z* transformation was applied in generated FC brains to further improve normal distribution and facilitate subsequent statistical analysis.

### Causal Connectivity Analysis

In the present work, GCA, implemented in Resting-State fMRI Data Analysis Toolkit (REST)^7^ and as one of the effective methods for inferring causal relationships, was applied to measure the CC between the two time series based on the previous researches (Wang et al., 2015). We first extracted time series of each subject from the SN subnetwork masks mentioned above and voxel in the brain, respectively, and the CC result can be then obtained using GCA. A brief introduction of GCA based on coefficient is provided below. For two given rs-fMRI series *x*(*t*) and *y*(*t*), supposing that it is more accurate to predict *x*(*t*) using the past time points of *x*(*t*) and *y*(*t*) than to predict *x* using *x*, then there exists a causal relationship between *x* and *y*, where *y* is called the cause and *x* is the effect. This is also analogous to other case analysis. The mathematical formula is constructed in the following form:

$$x\,\left(t\right)=a_{x,0}+\sum_{i=1}^{p}a_{x\,x,i}\,x\,\left(t-i\right)+\sum_{i=1}^{p}a_{y\,x,i}\,y\,\left(t-i\right)+$$

$$\sum_{i=1}^{p}b_{x,i}\,z_{j}\,\left(t-i\right)+\xi_{x}\,\left(t\right)$$

$$y\,\left(t\right)=a_{y,0}+\sum_{i=1}^{p}a_{x\,y,i}\,x\,\left(t-i\right)+\sum_{i=1}^{p}a_{y\,y,i}\,y\,\left(t-i\right)+$$

(1) $$\sum_{i=1}^{p}b_{y,i}\,z_{j}\,\left(t-i\right)+\xi_{y}\,\left(t\right)$$

where *p* is the model order to measure the lag of time series, and it was set to 1 in our work; **ξ** represents forecast error regression coefficient. *a*_*xx*_ and *a*_*yy*_ are the autoregressive coefficient, whereas *a*_*xy*_ and *a*_*yx*_ are regression coefficients that we used in our work; *z* denotes noise signal, and the covariate effect coefficient is denoted by *b*. In consequence, the problem mentioned above aimed to explore the CC alterations between the selected SN subnetworks and the whole brain across three groups.

## Results

### Demographic and Neurocognitive Characteristics

The demographic and neurocognitive data of all subjects are summarized in Table 1. One-way ANOVA presented the significant differences on age (*F* = 8.248, *p* = 0.016), Mini-Mental State Examination (MMSE) score (*F* = 9.129, *p* < 0.01), and CDR score (*F* = 68.98, *p* < 0.01). Nonetheless, it showed no significant difference on gender (*F* = 2.026, *p* = 0.139). Whereas lower MMSE scores indicate a greater degree of cognitive impairment, higher CDR scores show greater dementia. For the MMSE scores, the order from high to low was as follows: HC group (29.14 ± 1.49), SCD group (28.94 ± 0.83), and aMCI group (26.87 ± 2.72). Compared to the HC group (0.03 ± 0.11), the CDR scores increased in the SCD group and the aMCI group successively.

**TABLE 1:** Demographics and clinical measures of HC, SCD, and aMCI groups.

**Group**	**HC (*n* = 27)**	**SCD (*n* = 20)**	**aMCI (*n* = 28)**	***p***
Gender, female/male	20/7	10/10	10/18	0.139^a^
Age (years)	72.63 ± 4.50	72.38 ± 5.31	69.71 ± 7.26	0.016^b^
MMSE scores	29.14 ± 1.49	28.94 ± 0.83	26.87 ± 2.72	<0.01^b^
CDR scores	0.03 ± 0.11	0.12 ± 0.22	0.52 ± 0.10	<0.01^b^

**Numbers are given as means ± standard deviation (SD) unless otherwise stated. MMSE, Mini-Mental State Examination; CDR, Clinical Dementia Rating. ^*a*^The p-values were obtained by χ^2^-test. ^*b*^The p-value was obtained by one-way ANOVA.**

### Identified Regions of SN Subnetworks Using ICA

The SN subnetworks, including aSN and pSN networks, were extracted by ICA of all subjects. Spatial correlations of ICA indicated that the 34th component (*r* = 0.34) was the component most closely related to the aSN network; similarly, the 14th component (*r* = 0.36) was the component corresponding to the pSN. Subsequently, we obtained five clusters within the aSN and six clusters within the pSN of all subjects using one-sample *t*-test separately, consisting of two clusters of right IL, left IL, right supplementary motor area (SMA), left middle frontal gyrus (MFG), right MFG, left superior temporal gyrus (STG), right supramarginal gyrus (SG), left SG, right middle cingulum, and left pre-cuneus (PreCU), respectively (*p* < 0.05, TFCE-FWE corrected, cluster size > 10 voxels) (Table 2).

**TABLE 2:** Five significant clusters of the aSN and six significant clusters of pSN using one-sample *t*-test, respectively.

**Subnetwork**	**Region**	**Peak/MNI**			***t*-Score**	**Cluster size**
		***x***	***Y***	***z***		
aSN	R IL	39	12	0	6.1223	69
	L IL	−51	12	−3	7.4987	45
	R SMA	3	9	57	20.2433	809
	R MFG	30	39	30	6.6647	73
	L MFG	−30	45	30	4.8756	65
pSN	R IL	39	−9	−9	8.4505	40
	L STG	−36	−12	−9	5.9294	25
	R SG	60	−36	27	20.1412	266
	L SG	−60	−30	27	20.4620	339
	R MC	12	−33	45	8.107	20
	L PreCU	−6	−54	57	11.2489	29

**The x, y, and z coordinates are the primary peak locations in the MNI space. p < 0.05, TFCE-FWE corrected, cluster size > 10 voxels. L, left; R, right; IL, insula lobule; SMA, supplementary motor area; MFG, middle frontal gyrus; STG, superior temporal gyrus; SG, supramarginal gyrus; MC, middle cingulum; PreCU, pre-cuneus.**

### Altered FC Patterns of SN Subnetworks in the SCD and aMCI Groups

In the aSN, one-way ANOVA revealed four distinct clusters within the brain of three groups, including the left cerebellum superior lobule (CSL), left inferior temporal gyrus (ITG), right orbital inferior frontal gyrus, right lingual gyrus. Besides, compared to HC group within the mask after ANOVA, the aSN connected to the whole brain were separately increased in left orbital superior frontal gyrus, left IL, right caudate lobule (CL), left rolandic operculum gyrus (ROG), whereas decreased FC was found in the left CSL and left middle temporal gyrus (MTG) using two-sample *t*-test. Compared to the SCD group, the aMCI group exhibited decreased FC in the left MTG. Notably, compared to the HC group, decreased and increased FCs were both found in the SCD group, whereas no significant differences were found in the aMCI group (TFCE-FWE corrected, cluster size ≥ 10 voxels, *p* < 0.05). Moreover, the influences of age and gender were controlled in all of the results. At last for the pSN, we found no obvious differences at the 0.05 level using two-sample *t*-test (Table 3).

**TABLE 3:** The significant differences in FC in aSN network.

**Region**	**Peak/MNI**			***t*-Score**	**Cluster size**
	***x***	***y***	***z***		
**ANOVA**					
L cerebellum superior lobule	−45	−72	−30	7.063	462
L inferior temporal gyrus	−57	−54	33	10.8394	3060
R orbital inferior frontal gyrus	30	18	−24	11.4223	908
R lingual gyrus	−3	−63	6	7.7562	265
**SCD > HC**					
L orbital superior frontal gyrus	−24	42	−15	4.2171	29
L insula lobule	−33	12	6	3.7921	127
R caudate lobule	12	18	−9	3.9469	22
L rolandic operculum gyrus	−42	−6	12	3.8651	42
**HC > SCD**					
L cerebellum superior lobule	−51	−66	−39	3.6833	25
L middle temporal gyrus	−57	−9	−24	3.893	114
**aMCI > SCD**					
L middle temporal gyrus	30	18	−24	4.6672	42

**The x, y, and z coordinates are the primary peak locations in the MNI space. Cluster size > 200 voxels in one-way ANOVA, p < 0.05; cluster size > 10 voxels in two-sample t-test, p < 0.05, TFCE-FWE corrected. L, left; R, right.**

### Altered CC Patterns of SN Subnetworks in SCD and aMCI Groups

At first, we assumed that the selected aSN network was the cause, and the whole brain was the effect to explore the altered CC patterns between the aSN and the whole brain. The ANOVA demonstrated that the prominent differences have focused on the regions of right cerebellum inferior lobule (CIL), left CSL and right superior parietal lobule (SPL), respectively. In comparison with the HC group, the SCD group showed increased CC in the right SPL, whereas aMCI group exhibited decreased CC in the right CIL region. It is worth noting that we found no prominently significant differences within the brain between the aMCI and SCD groups in aSN (TFCE-FWE corrected, cluster size ≥ 10 voxels, *p* < 0.05) (Table 4).

**TABLE 4:** The significant differences in CC in aSN network when the selected aSN network is the cause and the whole brain is the effect.

**Region**	**Peak/MNI**			***t*-Score**	**Cluster size**
	***x***	***y***	***z***		
**ANOVA**					
R cerebellum inferior lobule	−6	−54	−51	8.2648	296
L cerebellum superior lobule	−15	−60	−18	7.4053	243
R superior parietal lobule	30	−72	57	12.7877	251
**SCD > HC**					
R superior parietal lobule	48	−51	54	4.8878	198
**HC > aMCI**					
R cerebellum inferior lobule	−6	−57	−51	4.063	165

**The x, y, and z coordinates are the primary peak locations in the MNI space. Cluster size > 200 voxels in one-way ANOVA, p < 0.05; cluster size > 10 voxels in two-sample t-test, p < 0.05, TFCE-FWE corrected. L, left; R, right.**

We next presumed that the whole brain was the cause, and the selected aSN network was the effect. The cluster of left CSL was given by the ANOVA. We found that compared to the HC group the SCD group exhibited decreased CC in the region of the right CIL, yet increased CC in the aMCI group. Further, compared to the SCD group, the aMCI group exhibited no prominent differences within the brain in aSN (TFCE-FWE corrected, cluster size ≥ 10 voxels, *p* < 0.05) (Table 5).

**TABLE 5:** The significant differences in CC in aSN network when the whole brain is the cause and the selected aSN network is the effect.

**Region**	**Peak/MNI**			***t*-Score**	**Cluster size**
	***x***	***y***	***z***		
**ANOVA**					
L cerebellum superior lobule	42	−36	−33	10.1827	680
**HC > SCD**					
R cerebellum inferior lobule	15	−30	33	−3.9058	519
**aMCI > SCD**					
R cerebellum inferior lobule	27	−54	−33	3.6655	32

**The x, y, and z coordinates are the primary peak locations in the MNI space. Cluster size > 200 voxels in one-way ANOVA, p < 0.05; cluster size > 10 voxels in two-sample t-test, p < 0.05, TFCE-FWE corrected. L, left; R, right.**

**FIGURE 1:**
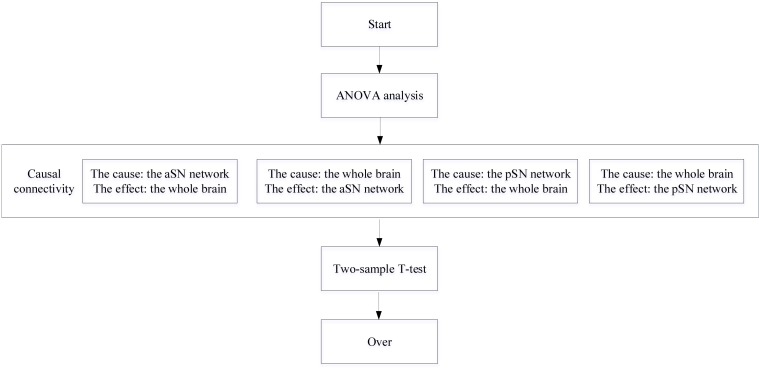
A flowchart depicting the CC process.

Then, we supposed that the selected pSN was the cause, and the whole brain was the effect. The ANOVA exhibited prominently significant differences in the right ITG, right inferior parietal lobule (IPL), right angular gyrus (AG), and right SMA, respectively. In comparison with the HC group, the SCD group exhibited decreased CC in the left IPL, left post-central gyrus (PG), and right AG, whereas the aMCI group exhibited decreased CC in right SMA. Compared to the SCD group, the aMCI group exhibited decreased CC in the left PreCU, corpus callosum (CCA), and right SMA. Interestingly, the SCD and aMCI groups all showed decreased CC in aSN when compared to the HC group (TFCE-FWE corrected, cluster size ≥ 10, *p* < 0.05) (Figure 1 and Table 6).

**TABLE 6:** The significant differences in CC in pSN network when the selected pSN is the cause and the whole brain is the effect.

**Region**	**Peak/MNI**			***t*-Score**	**Cluster size**
	***x***	***y***	***z***		
**ANOVA**					
R inferior temporal gyrus	48	−9	−39	8.0983	212
R inferior parietal lobule	−24	−57	36	9.5386	858
R angular	39	−63	36	9.4243	308
R supplementary motor area	9	−24	60	10.0099	282
**HC > SCD**					
L inferior parietal lobule	−24	−57	36	4.0374	30
L post-central gyrus	−39	−34	36	3.7147	13
R angular gyrus	39	−63	36	4.0322	42
**HC > aMCI**					
R supplementary motor area	0	−27	63	4.0201	85
**SCD > aMCI**					
L pre-cuneus	−18	−45	9	3.8167	21
Corpus Callosum	−15	−39	24	3.3527	49
R supplementary motor area	3	−21	63	3.7881	10

**The x, y, and z coordinates are the primary peak locations in the MNI space. Cluster size > 200 voxels in one-way ANOVA, p < 0.05; cluster size > 10 voxels in two-sample t-test, p < 0.05, TFCE-FWE corrected. L, left; R, right.**

And finally, we assumed that the whole brain was the cause, and the selected pSN was the effect. The ANOVA showed a statistically significant difference in the left STG. By comparison with the HC group, we found the regions of decreased CC in SG in the SCD group, whereas the aMCI group showed decreased CC and increased CC in the left IPL and left ROG, respectively. It is interesting that decreased CC was found in IPL, yet increased CC in ROG. Compared to the SCD group, the aMCI group exhibited a significant difference of increased CC in left post-central gyrus (LPG). It is noticeable that all of the results have been controlled for the influences of age and gender (Table 7).

**TABLE 7:** The significant differences in CC in pSN network when the whole brain is cause and the selected pSN is the effect.

**Region**	**Peak/MNI**			***t*-Score**	**Cluster size**
	***x***	***y***	***Z***		
**ANOVA**					
L superior temporal gyrus	−45	−39	24	12.0664	606
**HC > SCD**					
Supramarginal gyrus	−36	−39	30	3.8585	17
**HC > aMCI**					
L inferior parietal lobule	−39	−48	39	3.4764	20
**aMCI > HC**					
L rolandic operculum gyrus	−45	−39	24	4.8525	137
**aMCI > SCD**					
L post-central gyrus	−45	−18	27	3.8486	12

**The x, y, and z coordinates are the primary peak locations in the MNI space. Cluster size > 200 voxels in one-way ANOVA, p < 0.05; cluster size > 10 voxels in two-sample t-test, p < 0.05, TFCE-FWE corrected. L, left; R, right.**

## Discussion

We mainly aimed to explore the alteration patterns of FC and CC of the aSN and pSN networks to the whole brain in the aMCI and SCD groups and to investigate how this altered the regions of FC and CC to cognitive function. The novel aspect of our research is that we demonstrated the FC and CC alterations of SN subnetworks in the SCD and aMCI groups, accompanied by significant brain region analysis. Our results support the hypothesis that was put forward before to a great extent, and the research in this work also provides a new way to understand the stages of aMCI and SCD.

Over the years, the analysis in altered FC and CC of SN and other networks has been a research hotspot, but rarely investigations in SN subnetworks connected with the whole brain. Consistent with previous studies, the hub regions of aSN and pSN networks in this article using ICA, such as IL and cingulum, have been frequently reported to be the major hub regions of SN (Seeley et al., 2007; Menon and Uddin, 2010; Gao et al., 2020a, b, Wang et al., 2020). Then, for FC analysis, we found an interesting phenomenon that only aSN network showed the altered FC using a two-sample *t*-test across groups, proving that aSN might have more disruptive FC when compared to pSN. Notably for aSN, the patients with SCD have more brain regions with remarkable abnormalities than the aMCI group. Compared with the HC group, the patients with SCD have prominently altered CC in both left IL and right CL, which is consistent with previous studies that IL and CL are activated simultaneously (Postuma and Dagher, 2006). Besides, combining the prior research that CL plays a critical role in the brain’s learning and memory system and IL is closely related to somatosensory and motor functions (Murray et al., 1984; Bick et al., 2019). Taken together, the altered CC in left IL and right CL where patients with SCD showed prominent differences within the brain might lead to abnormal cognitive functions such as auditory processing, somatosensory, motor, and memory. Specifically, the region where the SCD group patients showed decreased FC in the left MTG in SCD compared to the HC group was similar to that in the aMCI group compared to the SCD group, yet only the former FC was reduced, and the latter FC was increased. According to a previously reported study, the MTG brain region is primarily involved in verbal or semantic cognition and is also associated with oral short-term memory (Vandenberghe et al., 1996). Moreover, the MTG brain region of the AD group showed increased FC, and it has been proved that function involved in semantic knowledge extraction is preserved and may be owed to the compensation mechanism to address memory and cognitive impairment (Peters et al., 2009; Cha et al., 2013). Hence, the alteration in the FC of left MTG found in this study may be explained by a compensation mechanism that exists in the human brain, and left MTG’s compensation mechanism of SCD may be stronger than aMCI. Interestingly, patients with SCD primarily appeared to have a decreased FC in left CSL when compared to the HC group, whereas no significant difference was found in patients with aMCI. The cerebellum is involved in motor and balance as well as advanced cognitive functions according to previous research (Gottwald et al., 2003), suggesting that the cerebellar-related cognitive functions of SCD might be subject to a potential effect inferred by the altered FC of aSN to the whole brain. A previous study has indicated that the SN is mainly responsible for cognition-related aspects and is the key interface for the cognitive system (La Corte et al., 2016). Meanwhile, according to the previous relevant studies, there exists obvious cognitive impairment in patients with aMCI when compared to SCD (Yan et al., 2018). Consequently, our results reveal that there might be a different impairment in FC of aSN in cognitive function across the aMCI and SCD.

A recent study has indicated that within-SN CC between the dACC and the striatum is abnormal in aMCI when compared to the HC group (Yu et al., 2019), yet there are only a few studies on the altered CC patterns between the SN subnetworks (aSN and pSN networks) and the whole brain. Then, a previous study has proven that directed connectivity, implemented in GCA within DPABI, can reveal the compensatory or pathological mechanisms of AD to some extent (Menon, 2011). Thus, in this follow-up, we analyzed the alterations of directed CC between the SN subnetworks and the whole brain. Compared to the HC group, in patients with aMCI, it was shown that aberrant connections are separately involved in the right CIL, right SMA, and left ROG, whereas patients with SCD exhibited more aberrant connection regions, comprising the right SPL, right CIL, left IPL, left PG, and right AG. Except for the right CIL region, the regions of significant difference between aSN and pSN were all different, proving that there might exist different communications for information between the SN subnetworks and other brain regions. Compared to the HC group, patients with SCD showed increased CC in the right CIL and left PG, whereas patients with aMCI showed decreased CC in the left PreCU, CCA, and right SMA. The PreCU is associated with many high levels of cognitive functions, such as episodic memory and the processing of self-related information (Herbet et al., 2019). The CCA is mainly connected with motor language center, bilateral visual hearing center, and so on, which is the communication channel of bilateral cerebral hemisphere cognitive function (Prendergast et al., 2018). In addition, PG is located in the parietal lobe of the cerebral cortex, between the central sulcus and the central posterior sulcus, corresponding to the somatosensory center (Yoshino et al., 2017). Thus, the aberrant CC in this article indicates that both the SCD group and aMCI group have different degrees of cognitive impairment, which is consistent with the findings of a previous study (Yan et al., 2018), and the altered CC may be affected by the brain’s compensation mechanism. Our research suggests, whether the aSN is the cause or effect, both exhibited statistical differences in the right CIL region, and no prominent difference for pSN was found. Also, no matter the pSN is the cause or effect, both showed statistical differences in the left PG region, and no difference for aSN was seen. Therefore, according to the aforementioned cerebellum involved in motor and balance, as well as advanced cognitive functions, and the PG involved in the somatosensory center, it can be deduced that CIL and left PG are sensitive and might be used as neuroimaging biomarkers to distinguish the cognitive function impairment of aSN and pSN. Interestingly, we also find that the altered CC of pSN is found prominently outnumbering that of aSN, signifying that pSN may have far more serious functional impairment and more compensation requirements and can be used as neuroimaging biomarkers for diagnosis of the early preclinical AD.

In conclusion, our findings show that both the FC and CC of the SN subnetworks (aSN and pSN) are distinctively disruptive in the early preclinical stages of AD consisting of SCD and aMCI. Moreover, the prominent difference in the distribution of aSN and pSN varies considerably, which may be used as neuroimaging biomarkers for diagnosis of the early preclinical AD.

## Conclusion

This study mainly reveals that the SCD and aMCI groups exhibit distinct alternations in aSN and pSN networks compared to the HC group. It turns out that the altered FC and CC in SCD and aMCI groups may reflect the changes in cognitive function, and there may be a compensation mechanism. Further, the sensitive neuroimaging biomarkers found in the FC and CC of SN subnetworks may provide new insight for the early detection of AD.

## Data Availability Statement

Publicly available datasets were analyzed in this study. This data can be found here: http://adni.loni.usc.edu/.

## Author Contributions

CH, CY, and HL guided experiments and correspondingly proposed some thesis writing strategies for the manuscript. EN, XH, FR, and DH checked for grammatical errors of manuscript, and discussed difficult problems in manuscript with CC.

## Conflict of Interest

The authors declare that the research was conducted in the absence of any commercial or financial relationships that could be construed as a potential conflict of interest.

## Footnotes

1 http://adni.loni.usc.edu/

2 http://restfmri.net/forum/RESTplusV1.2

3 http://www.mathworks.com/products/matlab/

4 https://www.fil.ion.ucl.ac.uk/spm/

5 http://rfmri.org/dpabi

6 http://icatb.sourceforge.net

7 http://www.restfmri.net/forum/REST_V1.8
